# In Vivo Dielectric Properties of Healthy and Benign Rat Mammary Tissues from 500 MHz to 18 GHz

**DOI:** 10.3390/s20082214

**Published:** 2020-04-14

**Authors:** Tuba Yilmaz, Fatma Ates Alkan

**Affiliations:** 1 Department of Electronics and Communication Engineering, Istanbul Technical University, Istanbul 34469, Turkey; 2 Department of Biophysics, Medical School, Beykent University, Istanbul 34520, Turkey; fatmaalkan@beykent.edu.tr

**Keywords:** in vivo dielectric properties, rat mammary tissues, DMBA-induced benign tumors

## Abstract

This work investigates the in vivo dielectric properties of healthy and benign rat mammary tissues in an attempt to expand the dielectric property knowledge of animal models. The outcomes of this study can enable testing of microwave medical technologies on animal models and interpretation of tissue alteration-dependent in vivo dielectric properties of mammary tissues. Towards this end, in vivo dielectric properties of healthy rat mammary tissues and chemically induced benign rat mammary tumors including low-grade adenosis, sclerosing adenosis, and adenosis were collected with open-ended coaxial probes from 500 MHz to 18 GHz. The in vivo measurements revealed that the dielectric properties of benign rat mammary tumors are higher than the healthy rat mammary tissues by 9.3% to 35.5% and 19.6% to 48.7% for relative permittivity and conductivity, respectively. Furthermore, to our surprise, we found that the grade of the benign tissue affects the dielectric properties for this study. Finally, a comparison with ex vivo healthy human mammary tissue dielectric properties revealed that the healthy rat mammary tissues best replicate the dielectric properties of healthy medium density human samples.

## 1. Introduction

Different animal tissues have been studied in the literature to both establish dielectric property models and to understand the dielectric property behavior of different tissue types. Although these studies reported a well established body of dielectric property knowledge for variety of tissues, there is still a need to expand this knowledge to understand disease-dependent in vivo dielectric property behavior of tissues and to enable animal testing of microwave medical devices.

Initial studies on animal tissues at microwave frequencies were performed to evaluate the effect of measurement conditions, death, and species variations [1,2,3]. More recently, reported studies include a large number of tissue types collected from rat, mouse, cattle, and swine under various measurement conditions for different applications. In one study, dielectric property change for various rat tissues with respect to age were reported [4]. In [5], ultrawideband dielectric properties of healthy swine liver, swine muscle, swine fat, and blood at increasing temperatures were reported with the motivation to enable microwave ablation and hyperthermia applications. In [6,7], effect of tissue hydration to dielectric properties was explored by performing experiments on rat muscle, rat fat, and mouse kidney tissues. In another study [8], dielectric properties of abdominal and thoracic tissues of cattle were investigated to enable research on wireless body area networks. In [9,10], rat skin and swine skin dielectric properties were characterized for testing of implantable antennas. In another study, the dielectric properties of healthy and diseased rat liver was studied to enable microwave biopsy and surgical margin detection applications [11]. Lastly, the in vivo and ex vivo average dielectric properties of breast carcinoma xenografted on nude mouse, mouse muscle, and mouse mammary fat pat between 1–5 GHz was reported in [12].

The reported studies were mostly focused on the healthy animal tissues since it requires resources, time, and strong interdisciplinary collaboration to form certain diseases, such as mammary carcinoma, in animal models. Therefore, dielectric spectroscopy research for breast cancer was mostly focused on dielectric property characterization of healthy and diseased samples obtained from breast reduction and cancer resection surgeries. In [13,14,15], ex vivo dielectric properties of diseased and healthy human breast have been studied. A comparison between in vivo and ex vivo samples obtained from cancer resection surgeries was given in [16]. The reported studies formed the basis of breast cancer imaging, treatment, and other diagnostic research. Conventional testing route for medical device development is to first perform preliminary tests on phantom models, next to employ the animal models and finally to test the devices on humans with clinical trials. The phantom materials are widely used in microwave medical device development as proof of concept. Despite recent employment of diseased animal models for microwave medical device testing [11,12], the reported studies are very limited and do not explore variety of animal and disease models. Therefore, there is still a need to study the dielectric properties of diseased tissues in animal models to form the dielectric property knowledge. To this end, this study reports in vivo dielectric property measurements of rat healthy mammary and benign tissues to,

- analyze the effect of breast tissue alteration on the in vivo dielectric properties of mammary tissue in rats,
- report healthy and diseased rat mammary tissue in vivo dielectric properties to enable the optimization of the microwave medical devices for testing with experimental animals.

Measured dielectric propertied are represented with fitted Cole–Cole models. The remainder of this paper is organized as follows: Section 2 describes the induction of tumors to animal models along with the protocols followed during the dielectric property collection and the methodology for Cole–Cole fitting. Section 3 gives the measured dielectric properties as well as the mathematical models fitted to the mean of the measured data. Comparison with literature data is given in Section 3.4. The conclusions are drawn in Section 4.

## 2. Material and Methods

### 2.1. Animals

Five female Sprague–Dawley experiment rats were obtained from Bogazici University, Center for Life Sciences and Technologies. At 47 days old four animals were given a single dose of carcinogenic solution via oral gavage. Carcinogenic solution was prepared by dissolving 20 mg/kg DMBA in 1 mL olive oil in an ultrasound bath. The solutions were freshly prepared and were given to the animals to trigger the formation of mammary tumors in the experiment animal. Twenty-four hours before the application, animals’ access to standard pellet food were restricted. After the application, the animals were left to rest for two days. Then the animals were weighed and checked weekly by hand for tumor formation. Throughout the experiment, the animals were kept in 12 h light/dark cycle and given ad libitum access to standard pellet food and tap water. One control animal was given 1 mL of olive oil as a dummy solution via oral gavage under the same conditions.

### 2.2. Measurement Setup and Procedure

**Figure 1:**
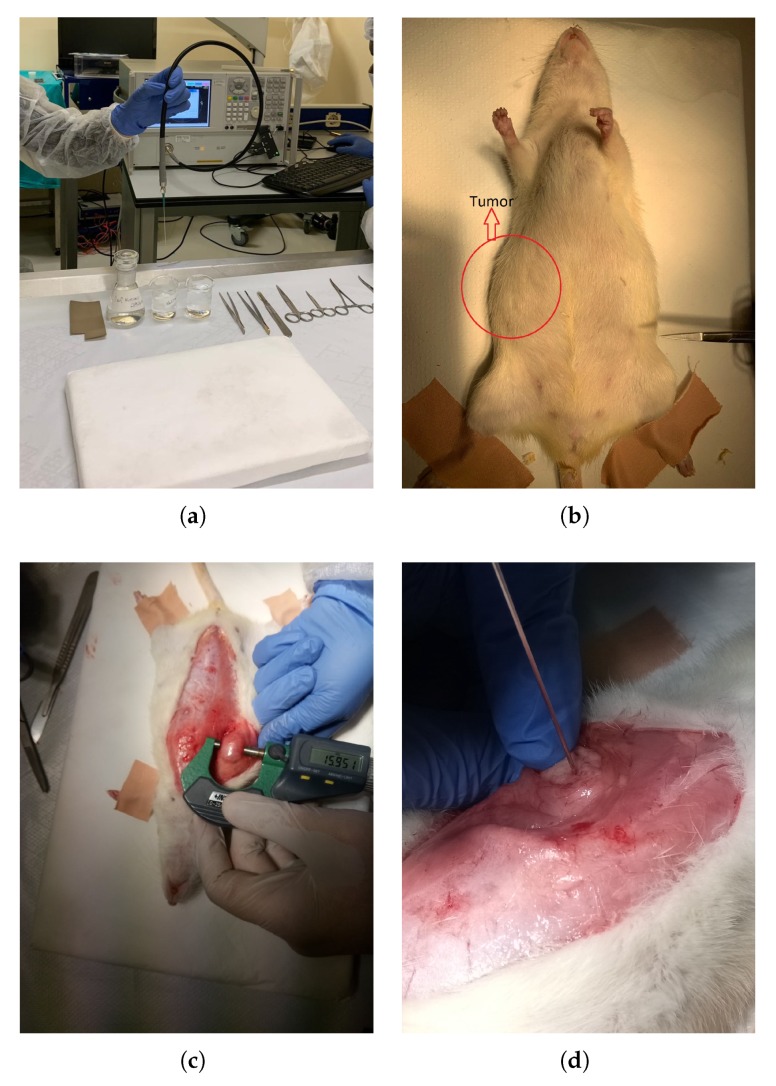
Protocol followed during in vivo dielectric property measurements of rat mammary tissues: (**a**) Measurement setup along with the network analyzer, RF cable, and open-ended coaxial probe; (**b**) A picture of the tumor gross view; (**c**) Exposed rat benign tumor with calliper measurement; (**d**) Tumor measurement with open-ended coaxial probe.

**Figure 2:**
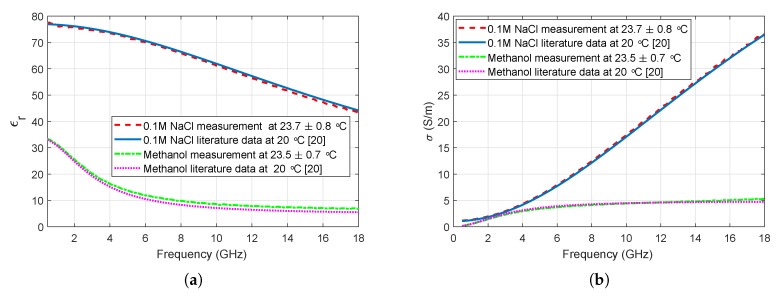
Comparison of measured reference liquids to literature data [20]: (**a**) Comparison of relative permittivity, (**b**) Comparison of conductivity.

The measurements were collected with the N5230A PNA Series Network Analyzer and commercial Agilent slim form probe kit using the 85070E software. The slim form probe has an aperture diameter of 2.2 mm. The sensing depth of the custom made probe with 2.2 mm aperture is reported to be between 0.75 and 1.5 mm in homogeneous materials [17]. Similarly, the sensing depth of the slim form probe is reported to be between 1.05 and 1.41 mm for homogeneous materials [18]. For heterogeneous two layered high dielectric property contrast materials, the sensing depth is reported to be well below 1 mm [18]. Agilent 20 GHz flexible RF cable was used to connect the probe to the analyzer. During the measurements, the probe was held by the experimenter. A picture of the measurement setup is given in Figure 1a. Before the measurements, the probe kit was calibrated with open, short, and distilled water following the standard calibration procedure [19]. During calibration, the average distilled water temperature was 24.6 ± 0.7 °C. After performing each calibration, the calibration validity was checked by measuring the dielectric properties of known liquids. Methanol and 0.1 M NaCl solution were used as known liquids since their dielectric properties are well documented in the literature [20]. Comparisons between the measurements and literature data [20] are given in Figure 2.

The animals were given a mixture of 80 mg kg^−1^ ketamine and 10 mg kg^−1^ xylazine via intraperitoneal injection after the calibration was completed. An anesthetized experiment animal picture along with a gross view of a tumor is given in Figure 1b. When the animal was fully anesthetized, the skin on the peritoneal region was incised. An incised experiment animal with a tumor is shown in Figure 1c. Then based on the tumor formation, dielectric properties were collected either from the tumor or healthy tissue. Five measurements were taken from each measurement point. Measurements were taken from four to ten points from tumors based on the formation of the tumor. Measurements from healthy samples were collected from four healthy samples using the same control group animal. Five consecutive measurements were collected from each sample. It should be noted that during the tumor measurements the sample was exposed without removing it from the main site. The tumors were located in the mammary tissue; thus, they were easily accessed after peritoneal incision. The authors did not disintegrate the tissue under test. A close-up view of the in vivo dielectric property measurement is given in Figure 1d. The calibration was renewed after every 20 in vivo measurements; that is, every 30 to 40 min. Weights of the animals ranged from 244 to 292 g at the time of the experiments. The ages of the animals ranged from 336 to 446 days old at the time of the experiments. The average tissue temperature at the measurement point was 31.5 ± 1.8 °C. After the completion of the measurements, the experiment animal was sent for euthanasia and the tissue under test was removed. The resected tissues were preserved in a 10% formalin solution for pathological analysis.

### 2.3. Measurement Samples

Three out of four animals developed tumors that were detectable via hand examination. The dimensions of the tumors and the pathological evaluations are given in Table 1. During dielectric property measurements, sample 4 was filled with liquid and it was unintentionally drained. Due to the limited amount of collected data, measurements taken from sample 4 are excluded from this study. Note that the pathological examination did not reveal any anomaly in sample 4. According to the pathological examination results, all remaining tumors were benign. The tumor grades obtained from pathology along with the samples’ dimensions are given in Table 1.

### 2.4. Mathematical Model

The Cole–Cole expression, given in Equation (1), is used in the literature to represent the dielectric property behavior of frequency dispersive materials over high frequency range. The variables in Cole–Cole equation $\epsilon_{\infty }$ is the dielectric constant at very high frequencies, $\epsilon_{s}$ is the static dielectric constant, *τ* is the relaxation time, *α* is the broadening parameter of the relaxation, and $\sigma_{i}$ is the ionic conductivity of the sample. These variables are named Cole–Cole parameters. The Cole–Cole parameters depend on the dielectric property behavior of the material under test. In this work, the parameters are fitted with Particle Swarm Optimization (PSO) algorithm, the details of the technique is described in [11,21]; therefore, here it will be briefly described.

(1) $$\epsilon^{{}^{\prime }}-i\epsilon^{{}^{\prime \prime }}=\epsilon_{\infty }+\frac{\epsilon_{s}-\epsilon_{\infty }}{1+{\left(iw\tau \right)}^{\left(1-\alpha \right)}}+\frac{\sigma }{iw\epsilon_{0}}$$

where $\epsilon^{{}^{\prime }}$ is the relative dielectric constant namely relative permittivity, $\epsilon^{{}^{\prime \prime }}$ is dielectric loss factor, and *ω* is the angular frequency.

The PSO algorithm is a heuristic optimization technique and it searches a pre-defined domain to reach a solution. The pre-defined solution domain is constructed by defining intervals to the listed five Cole–Cole parameters. The solution domain defined for this study is given in Table 2. The PSO starts by randomly evaluating solutions that meets the constraints, the solution is named a particle and it is stored as a vector. Fifty particles were used in this work; that is, fifty solutions are evaluated in a single iteration. Then, in each iteration particles updates their positions and velocity based on their personal scores and the global best score of the swarm. If a particle finds a solution outside the pre-defined domain, it is simply punished with a bad fitness value. The fitness value is calculated to rank the goodness of solutions and check if a solution meets a pre-defined criteria. The fitness function used in this study is Euclidean distance and it is given in Equation (2).

(2) $$error=\frac{1}{N}\sum_{i=1}^{N}\left[{\left(\frac{\epsilon_{w_{i}}^{{}^{\prime }}-{\hat{\epsilon }}_{w_{i}}^{{}^{\prime }}}{median(\epsilon_{w_{i}}^{{}^{\prime }})}\right)}^{2}+{\left(\frac{\epsilon_{w_{i}}^{{}^{\prime \prime }}-{\hat{\epsilon }}_{w_{i}}^{{}^{\prime \prime }}}{median(\epsilon_{w_{i}}^{{}^{\prime \prime }})}\right)}^{2}\right]$$

$\epsilon_{w_{i}}^{{}^{\prime }}$ and $\epsilon_{w_{i}}^{{}^{\prime \prime }}$ represents the measured dielectric properties at the frequency of interest, the dielectric properties calculated with the suggested solution are shown with ${\hat{\epsilon }}_{w_{i}}^{{}^{\prime }}$ and ${\hat{\epsilon }}_{w_{i}}^{{}^{\prime \prime }}$. If a solution reaches an Euclidean distance smaller than the threshold criteria the iteration stops. The threshold was set to 10^−3^ units in this study. The N is the number of points used in the measured frequency range, *N* = 179 for this study.

## 3. Results and Discussion

### 3.1. Measured In Vivo Dielectric Properties

**Figure 3:**
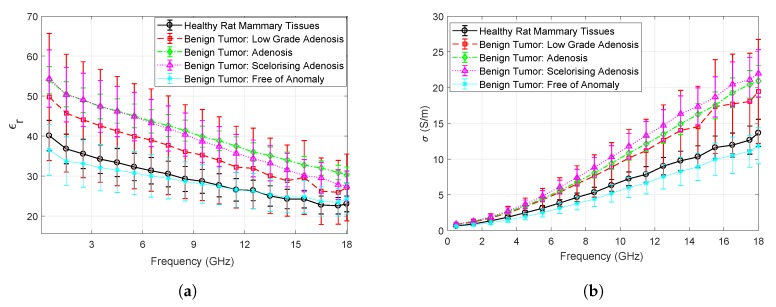
In vivo dielectric property measurement results of rat mammary tissues with error bars indicating the standard deviation: (**a**) Comparison of relative permittivity measurements collected from benign tumor and healthy tissues; (**b**) Comparison of conductivity measurements collected from benign tumor and healthy tissues.

Measured relative permittivity and conductivity results are given in Figure 3a,b. It can be seen that the dielectric properties of the benign tumor are dependent on the grade of the tumor in this study. Ultimately, a limited number of animals were studied in this work; therefore, variability between animals can not be reported at this stage. Grade, as well as the diagnosis of the tumor tissue, is assigned by the pathologist based on the cell differentiation from the healthy mammary tissues. A low-grade adenosis shows limited difference from healthy tissue whereas a sclerosing adenosis can show further differentiation from the healthy tissue in cellular level. On the macro level, an adenosis tissue is a lump that is formed from gland-like tissue and sclerosing adenosis is also a lump which contains gland- and scar-like tissues. Measured dielectric properties seem to reflect the changes between healthy, low-grade adenosis, and adenosis tissue both with relative permittivity and conductivity measurements for this work. A limited discrepancy at high frequencies emerges for both relative permittivity and conductivity between the adenosis and sclerosing adenosis tissue. These discrepancies are quantified for the whole frequency band with Equation (3).

(3) $$\%△\varepsilon =\frac{1}{N}\sum_{i=1}^{N}\left|\frac{\varepsilon_{i}-\bar{\varepsilon_{i}}}{\bar{\varepsilon_{i}}}\right|*100$$

where *N* is the number of measurement frequency points between 500 MHz to 18 GHz (*N* = 179). *ε* variable represents the relative permittivity or conductivity based on the target parameter.

The mean relative permittivity discrepancy between the healthy and benign tissues are 9.3%, 35.5%, and 31.4% for low-grade adenosis, adenosis, and sclerosing adenosis tissues, respectively. The mean conductivity discrepancy between the healthy and benign tissues are 19.6%, 44.3%, and 48.7% for low-grade adenosis, adenosis, and sclerosing adenosis tissues, respectively. The mean relative permittivity of low-grade adenosis is 24.0%, and 23.9% lower than adenosis and scelorising adenosis, respectively. Similarly, mean conductivity of low-grade adenosis is 20.8%, and 24.3% lower than adenosis and sclerosing adenosis, respectively. The mean relative permittivity of sclerosing adenosis is slightly lower than the adenosis at higher frequencies with 3.2% discrepancy between the two tissue types. The mean conductivity of sclerosing adenosis is 3.1% higher than adenosis tissue.

When relative permittivity behavior, given in Figure 3a, is analyzed, the relative permittivity of the healthy tissue is always lower than adenosis and sclerosing adenosis tissue below approximately 13 GHz. The discrepancy is also clear above 6 GHz for conductivity, given in Figure 3b. For the low-grade adenosis tissue the large standard deviation seems to suggest that the individual measurements can fall healthy tissue to higher grade benign tissue ranges. Ultimately, the mean of the low-grade adenosis measurements separates well from other tissues; however, a single measurement may not be representative of the mean. Therefore, it is worth noting that a single measurement cannot be safely distinguished from healthy or higher grade benign tissue.

### 3.2. Uncertainty Analysis

**Figure 4:**
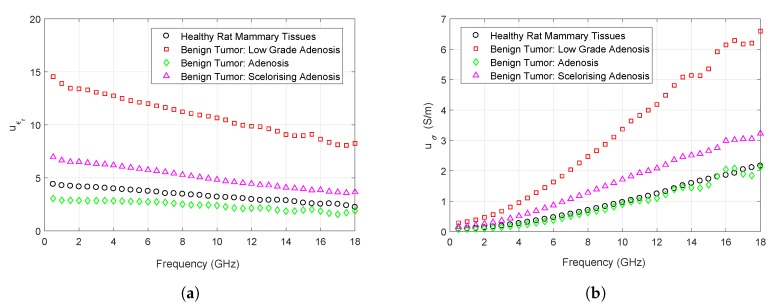
Calculated combined uncertainty values for rat mammary tissues: (**a**) Comparison of relative permittivity uncertainty calculations for benign tumor and healthy tissues; (**b**) Comparison of conductivity uncertainty calculations for benign tumor and healthy tissues.

Combined uncertainties of dielectric property measurements were calculated by combining the contributions of Type A ($u_{A}$) and Type B ($u_{B}$) errors shown in Equation (4) [6,22]. Type A errors refers to the random errors and Type B errors refers to the systematic errors. Type A error was calculated by using the repeated measurements taken from a sample where the standard deviation from the mean of the measurements were divided by the square root of the number of measurements. Type B error was calculated by finding the distance between minimum and maximum measurements and dividing the distance to square root of three. Mean combined uncertainty values for each tissue type are given in Table 3. Combined uncertainty values of each tissue type between 0.5 and 18 GHz are shown in Figure 4a,b for relative permittivity and conductivity, respectively.

(4) $$u=\sqrt{{u_{A}}^{2}+{u_{B}}^{2}}$$

A dielectric property comparison of diseased and healthy rat mammary tissues at 2.4 and 5.8 GHz, widely used frequencies in the Industrial, Scientific, Medical (ISM) band, are given in Table 4. The maximum difference between mean relative permittivity and conductivity of healthy and benign tissues at ISM bands are respectively, 12 units and 1.5 (S/m).

### 3.3. Cole–Cole Fitting

**Figure 5:**
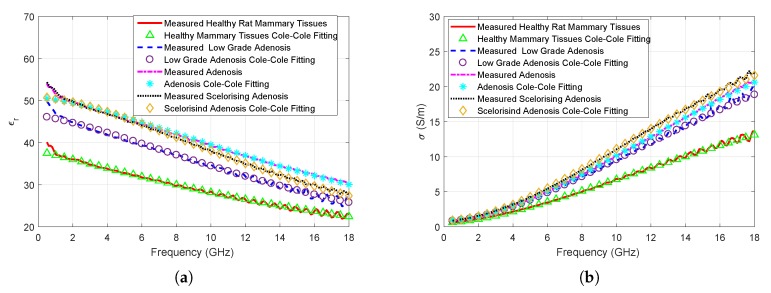
Comparison of the measured median dielectric properties and Cole–Cole fitting results: (**a**) Measured and Cole–Cole parameter relative permittivity values; (**b**) Measured and Cole–Cole parameter conductivity values.

The Cole–Cole parameters were fitted to the measured in vivo median dielectric properties of four breast tissue types. Comparisons between the data obtained with using the Cole–Cole parameters and measurements are shown in Figure 5a,b. As it can be seen from the figures and Euclidean distance values given in Table 5, the fitted parameters represents the measured dielectric properties with high accuracy. Calculated Euclidean distances were smaller than 10^−3^ units for all fittings. However, from 0.5 to 1.0 GHz the fittings do not fully represent the measured relative permittivities. This can potentially be resolved by using two-pole Cole–Cole equation. Nevertheless, the solutions given in Table 5 can be utilized to replicate the mean measurement values with high accuracy for healthy and benign mammary tissues from 1 to 18 GHz.

The solution to the Cole–Cole equation is not unique. Therefore, different combinations of the Cole–Cole parameters can satisfy the required threshold. Nevertheless, the relative permittivity discrepancy shown in Figure 3a is reflected in the $\epsilon_{s}$ variable given in Table 5. The conductivity discrepancy shown in Figure 3b is represented with the $\sigma_{i}$ (S/m) parameter for healthy and tumor tissues. However, the discrepancy is unclear when the $\sigma_{i}$ (S/m) parameter is compared for the benign tissues.

### 3.4. Comparison with the Reported Ex Vivo Human Breast Tissue Dielectric Properties

Rat mammary gland development and mammary carcinoma formation is similar to human mammary tissue alterations [23]; therefore, rats are frequently used as experiment animals for many different studies including but not limited to drug and toxicology research [24,25,26]. Despite these similarities, the size of the healthy human breast tissues differs from rat breast tissues due to the heterogeneous presence of adipose, glandular and fibroconnective tissues in large volumes when compared to rat breast tissues. Therefore, the dielectric properties of human breast categorization is represented in multiple classes based on the adipose tissue content [13,14,15]. To this end, there is a need to understand how closely the rat breast tissues are replicating the dielectric properties obtained from the human tissues.

**Figure 6:**
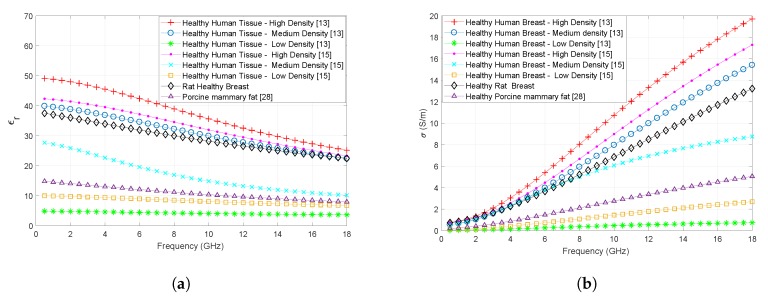
Dielectric property comparisons of ex vivo healthy human breast tissues and porcine mammary tissues with high, median, low density and healthy in vivo rat breast tissues: (**a**) Relative permittivity values; (**b**) Conductivity values.

In Figure 6a,b, measured in vivo dielectric property comparison of healthy rat breast tissues with the ex vivo human literature data [14,15] is given. When rat dielectric property data is compared with the data obtained from [14], the in vivo rat tissue relative permittivity and conductivity values are slightly lower than those collected from the medium density healthy breast tissue. The discrepancies are, respectively, 5.2% and 10.8% for relative permittivity and conductivity in the whole frequency band. When it is compared to the data reported in [15], the healthy rat breast dielectric properties are close to the ex vivo dielectric properties of the high density breast tissue with discrepancies of 12.9% and 18.6% for relative permittivity and conductivity, respectively. Dielectric properties of swine mammary fat tissue [27] are also given to provide interspecies comparison. It should also be noted that the dielectric properties reported in [14] were collected from 19 to 27 °C, in [15] 19 to 22 °C, and in [27] the dielectric properties are reported in 37 °C. In this study, the average tissue temperature was 31.5 ± 1.8 °C. The temperature is known to change the dielectric property behavior. Broadly, increase in temperature of biological tissue decreases the dielectric properties [5].

From these comparisons, we can conclude that the healthy rat tissue dielectric properties replicate the dielectric properties of fibroconnective and gland tissue with medium to low adipose content. These findings indicate that testing microwave medical devices with rat breast tissues can provide a good approximation of performances of such devices for human tissues.

**Figure 7:**
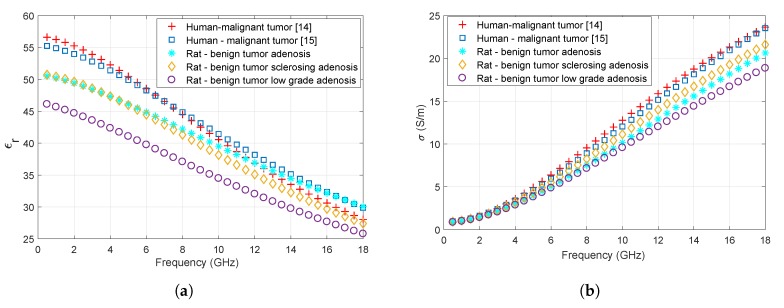
Dielectric property comparisons of ex vivo malignant human breast tumor tissues and in vivo rat benign breast tumor tissues: (**a**) Relative permittivity values; (**b**) Conductivity values.

Figure 7a,b shows the comparison of measured in vivo rat benign tumor with ex vivo human malignant breast tumor tissues [14,15] for relative permittivity and conductivity, respectively.

Relative permittivity discrepancy for the whole frequency band between the malignant human tumor values reported in [14] and rat low-grade adenosis, adenosis and sclerosing adenosis are 24.6%, 6.0%, 5.5%, respectively. Relative permittivity discrepancy for the whole frequency band between the malignant human tumor values reported in [15] and rat low-grade adenosis, adenosis and sclerosing adenosis are 32.1%, 6.6%, 10.0%, respectively. Conductivity discrepancy for the whole frequency band between the malignant human tumor values reported in [14] and rat low-grade adenosis, adenosis and sclerosing adenosis are 47.9%, 22.6%, 19.6%, respectively. Conductivity discrepancy for the whole frequency band between the malignant human tumor values reported in [15] and rat low-grade adenosis, adenosis and sclerosing adenosis are 41.2%, 17.0%, 14.1%, respectively. The dielectric property discrepancy between human fibroglandular tissues and malignant tumors is reported to be 10%. Here, the reported discrepancy between the rat benign tumors and malignant human tissues is lower than 10% for sclerosing adenosis and adenosis tissues and higher than 14% for sclerosing adenosis and adenosis tissues. The dielectric property discrepancy is above 24% for low-grade adenosis.

Microwave hyperthermia treatment aims to heat up the malignant tumors. The healthy tissues are used for modelling purposes during design of microwave treatment and diagnostic technologies. On the other hand, both malignant and benign tissue dielectric properties are essential for microwave diagnostic technologies. Healthy, benign, and malignant tissue dielectric properties are particularly useful for open-ended coaxial probe based diagnostics technologies [11,12]. Ultimately, there is a need to expand this study by collecting large scale in vivo dielectric properties from healthy, benign, and malignant rat mammary tissues.

## 4. Conclusions

Testing of microwave medical devices is limited to phantom materials and ideally such devices should be tested on animals before clinical research. Although the dielectric properties of ex vivo healthy human mammary tissues are well documented in the literature, knowledge on in vivo dielectric properties of healthy and diseased animal mammary tissues is limited. Such knowledge is essential when designing and testing microwave imaging, hyperthermia, and RF/microwave sensing devices for animal experiments. To enable testing of these devices with animals, there is a need to tabulate the in vivo dielectric properties of healthy and diseased animal tissues. The goal of this study is to report the dielectric properties of healthy and benign rat mammary tissues which opens up the possibility to optimize the design of microwave diagnostic and therapeutic devices for testing on animals. Additionally, this study reveals the in vivo dielectric property behavior of rat mammary tissues which can be used for understanding the in vivo dielectric property behavior of human tissues. To this end, dielectric properties of chemically induced benign tumors and healthy rat mammary tissues were collected from 500 MHz to 18 GHz in this study. To the best of the authors’ knowledge these properties have not been previously reported in the literature. It can be concluded that the in vivo dielectric property behavior of benign rat tissues differs from both healthy rat mammary tissues and also differs within itself. The grade of the adenosis tissue affects the dielectric properties and the benign human mammary tumor dielectric properties are expected to display similar trend. Further, a comparison with ex vivo dielectric properties of human heathy mammary tissues revealed that the dielectric properties of rat mammary tissues closely replicates the medium to high content glandular and fibroconnective human mammary tissue.

## Figures and Tables

**Table 1:** The dimensions of the samples, results obtained from pathological examination and the total number of measurements.

Sample	Height (mm)	Width (mm)	Depth (mm)	Number of Measurements	Pathological Analysis
Sample 1	14.7	18.6	4.4	50	Low-Grade Adenosis
Sample 2	16.0	17.9	5.2	40	Sclerosing Adenosis
Sample 3	10.3	14.4	3.29	20	Adenosis
Sample 4	4.5	5.4	3.1	5	Free of Anomaly
Healthy	2.3	N/A	N/A	20	Healthy Breast
Samples					Tissue

**Table 2:** Intervals chosen for the Cole–Cole parameters to define the solution domain.

Parameter	$\epsilon_{\infty }$	$\epsilon_{s}$	*τ* (ps)	*α*	$\sigma_{i}$ (S/m)
Min Value	1	1	1	0.01	0.01
Max Value	10	100	30	1	10

**Table 3:** Mean combined uncertainty for relative permittivity and conductivity of each tissue type.

Mean Combined Uncertainty	Healthy Rat Mammary Tissue	Low-Grade Adenosis	Adenosis	Sclerosing Adenosis
$u_{\epsilon_{r}}$	3.4	10.9	2.4	5.1
$u_{\sigma }$ (S/m)	1.0	3.2	0.9	1.6

**Table 4:** Mean of all in vivo dielectric property measurement comparisons collected from healthy and benign rat mammary tissues along with uncertainty values at Industrial, Scientific, Medical (ISM) bands.

Frequency (GHz)	Dielectric Properties	Healthy	Low-Grade Adenosis	Adenosis	Scelorising Adenosis
2.4	$\varepsilon_{r}$	36.3 ± 4.2	39.6 ± 13.3	48.7 ± 2.8	47.9 ± 6.4
	*σ* (S/m)	1.3 ± 0.2	1.4 ± 0.6	1.8 ± 0.1	1.8 ± 0.3
5.8	$\varepsilon_{r}$	32.3 ± 3.8	35.8 ± 12.1	44.0 ± 2.7	43.1 ± 5.8
	*σ* (S/m)	3.4 ± 0.4	3.9 ± 1.6	4.7 ± 0.3	4.9 ± 0.8

**Table 5:** One-pole Cole–Cole parameters fitted to the mean of the measured dielectric properties of healthy and benign rat mammary tissues.

Cole–Cole Parameters	Healthy	Low-Grade Adenosis	Adenosis	Scelorising Adenosis
$\epsilon_{\infty }$	4.05	2.83	5.11	5.40
$\epsilon_{s}$	38.07	46.50	50.79	50.94
*τ* (ps)	7.75	8.27	7.87	9.20
*α*	0.19	0.12	0.09	0.06
$\sigma_{i}$ (S/m)	0.60	0.81	0.89	0.88
*Euclidean Distance*	7.66 × 10^−4^	8.28 × 10^−4^	4.33 × 10^−4^	5.05 × 10^−4^
